# Characterization of Three Vasopressin Receptor 2 Variants: An Apparent Polymorphism (V266A) and Two Loss-of-Function Mutations (R181C and M311V)

**DOI:** 10.1371/journal.pone.0065885

**Published:** 2013-06-06

**Authors:** Stephen P. Armstrong, Ruth M. Seeber, Mohammed Akli Ayoub, Brian J. Feldman, Kevin D. G. Pfleger

**Affiliations:** 1 Laboratory for Molecular Endocrinology-G Protein-Coupled Receptors, Western Australian Institute for Medical Research and Centre for Medical Research, The University of Western Australia, Nedlands, Perth, Western Australia, Australia; 2 Protein Research Chair - Department of Biochemistry, College of Science, King Saud University, Riyadh, Kingdom of Saudi Arabia; 3 Pediatric Endocrinology, Department of Pediatrics, Stanford University, Stanford, California, United States of America; Aarhus University, Denmark

## Abstract

Arginine vasopressin (AVP) is released from the posterior pituitary and controls water homeostasis. AVP binding to vasopressin V2 receptors (V2Rs) located on kidney collecting duct epithelial cells triggers activation of Gs proteins, leading to increased cAMP levels, trafficking of aquaporin-2 water channels, and consequent increased water permeability and antidiuresis. Typically, loss-of-function V2R mutations cause nephrogenic diabetes insipidus (NDI), whereas gain-of-function mutations cause nephrogenic syndrome of inappropriate antidiuresis (NSIAD). Here we provide further characterization of two mutant V2Rs, R181C and M311V, reported to cause complete and partial NDI respectively, together with a V266A variant, in a patient diagnosed with NSIAD. Our data in HEK293FT cells revealed that for cAMP accumulation, AVP was about 500- or 30-fold less potent at the R181C and M311V mutants than at the wild-type receptor respectively (and about 4000- and 60-fold in COS7 cells respectively). However, in contrast to wild type V2R, the R181C mutant failed to increase inositol phosphate production, while with the M311V mutant, AVP exhibited only partial agonism in addition to a 37-fold potency decrease. Similar responses were detected in a BRET assay for β-arrestin recruitment, with the R181C receptor unresponsive to AVP, and partial agonism with a 23-fold decrease in potency observed with M311V in both HEK293FT and COS7 cells. Notably, the V266A V2R appeared functionally identical to the wild-type receptor in all assays tested, including cAMP and inositol phosphate accumulation, β-arrestin interaction, and in a BRET assay of receptor ubiquitination. Each receptor was expressed at comparable levels. Hence, the M311V V2R retains greater activity than the R181C mutant, consistent with the milder phenotype of NDI associated with this mutant. Notably, the R181C mutant appears to be a Gs protein-biased receptor incapable of signaling to inositol phosphate or recruiting β-arrestin. The etiology of NSIAD in the patient with V266A V2R remains unknown.

## Introduction

Arginine vasopressin (AVP) is released from the posterior pituitary and controls water balance homeostasis [1]–[3]. AVP, binding to arginine vasopressin V2 receptors (V2R) located on the basolateral membrane of kidney collecting duct epithelial cells, triggers activation of Gs proteins, leading to increased cAMP levels, and activation of Protein Kinase A [1]–[3]. This in turn causes trafficking of aquaporin-2 water channels to the apical membrane of collecting duct cells, resulting in increased water permeability and antidiuresis [1]–[3].

The V2R is a 7-transmembrane spanning G protein-coupled receptor (GPCR) [4], [5]. Typically, loss-of-function (in terms of cAMP signaling) V2R mutations cause nephrogenic diabetes insipidus (NDI), characterized by an inability to concentrate urine resulting in excessive urine production, dehydration and thirst [2], [3]. Administration of exogenous AVP fails to restore normal water balance [2], [3]. In contrast, gain-of-function mutations cause nephrogenic syndrome of inappropriate antidiuresis (NSIAD), characterized by inappropriately concentrated urine resulting in excessive sodium excretion, low plasma sodium concentration, and low serum osmolality [6]. A key feature is the absence of detectable levels of AVP in serum, whereas the closely related syndrome of inappropriate antidiuretic hormone secretion (SIADH) is typically associated with measurably elevated AVP levels and consequent V2R hyperactivity [6], [7].

There are now more than 200 different V2R mutations known to cause NDI [8], but only three that cause NSIAD [6], [9]. This is not surprising considering that many changes to a protein structure can result in impaired function, whereas a single point mutation able to constitutively activate a receptor is much more rare. Interestingly, the two opposing phenotypes of NDI and NSIAD can both result from distinct mutations at R137, which is present within the conserved DRY/H motif of the V2R, a region critical for stabilizing the receptor in either inactive or active conformations [10], [11]. Substitution of R137 with H results in NDI due to an inability of this mutant receptor to stimulate cAMP production [12]. Whether the loss-of-function is primarily due to G protein uncoupling, reduced forward trafficking and/or constitutive internalization is a matter of debate, and it is not inconceivable that all of these aspects of receptor pharmacology play a role in the overall phenotype [13], [14]. In contrast, substitution of the same residue with C or L favors constitutive activation and cAMP production, causing NSIAD [6]. Notably, both R137H and R137C/L mutants recruit β-arrestin constitutively and are constitutively internalized [13]–[16]. In contrast, the NSIAD-causing F229V mutation, although resulting in constitutive cAMP activation, does not result in constitutive β-arrestin interaction. Instead, this receptor is able to recruit β-arrestin in a ligand-dependent manner similar to that observed with wild-type V2R, indicating a degree of biased coupling [9].

Correct protein folding and trafficking to the plasma membrane are critical for V2R function. Indeed, it is thought that the majority of disease-causing mutations of the V2R, especially those associated with NDI, result in significant intracellular retention of the receptor due to a failure to pass quality control systems of the endoplasmic reticulum and early secretory pathway [2], [17], [18].

In this study we further characterize two previously reported mutations of the V2R from patients with NDI, R181C [19]–[25] and M311V [25], and a third potential mutation (V266A) from a patient with a putative diagnosis of NSIAD. V266A has been previously reported in combination with a nonsense mutation (W200X/V266A) in a patient with NDI [26], but mutation of V266A alone by those authors had no effect on cAMP accumulation (E_max_ or EC_50_), ELISA studies or radioligand binding. However, inositol phosphate production, β-arrestin interaction and receptor ubiquitination were not assessed. In the more recent case of a patient with symptoms of NSIAD, including hyponatremia and low ADH levels that presented during infancy, no variation in V2R was identified other than V266A. None of the patient's immediate family was clinically affected and as they declined genetic testing, unfortunately it is not known if they also possessed the same variation in V2R.

We have found that for the R181C and M311V mutant receptors, the degree of receptor activity lost (for cAMP, inositol phosphate accumulation and β-arrestin recruitment) correlated with the severity of disease. However, with the assays we have utilized, we could find no functional difference between wild-type and V266A receptor, even for inositol phosphate accumulation, β-arrestin interaction and receptor ubiquitination. Hence the etiology of NSIAD in this patient remains uncertain. Most interestingly, our analysis also revealed that R181C acts as a Gs protein-biased receptor, unable to couple to β-arrestin or stimulate inositol phosphate production, yet retaining some ability to increase intracellular cAMP.

## Materials and Methods

### Ethics Statement

The R181C and M311V mutations have been published previously [19]–[25]. We consulted the Stanford University Institutional Review Board (IRB) and they stated, in accordance with U.S. Code of Federal Regulations Title 45 part 46, that the V266A case should not be part of a protocol. V2R gene sequencing for the patient with the V266A variant was performed as a diagnostic test in order to guide clinical care and was performed in a CLIA approved clinical laboratory, with the sequence result being reported from the CLIA laboratory. The clinical laboratory also obtained written consent from the patient prior to performing genetic testing. There were no patient samples used in any of the research experiments presented in this manuscript.

### Materials

Wild-type and mutant HA-tagged V2R and V2R/Rluc8 cDNA constructs were generated by PCR amplification of receptor cDNA (with appropriate site-directed mutagenesis) and ligated into pcDNA3 containing the corresponding expression vectors, in a manner similar to that described previously [13]. Rluc8 cDNA was kindly provided by Andreas Loening and Sanjiv Gambhir (Stanford University, CA) [27]. Vasopressin receptor 2 (V2R)/Rluc8 was generated as described previously [13], [16]. The β-arrestin2/Venus cDNA construct was prepared previously [13] from pcC2-Venus kindly provided by Atsushi Miyawaki (RIKEN Brain Science Institute, Wako-city, Japan). Ubiquitin K48,63A/Venus was prepared as described [28]. Arginine Vasopressin (AVP) was from Sigma-Aldrich.

### Cell Culture and Transfection

HEK293FT (Life Technologies) and COS7 (ATCC) cells were maintained at 37°C in 5% CO_2_ and Dulbecco's modified Eagle's medium (DMEM) containing 0.3 mg/ml glutamine, 100 IU/ml penicillin and 100 µg/ml streptomycin (Gibco) supplemented with 10% foetal calf serum (FCS). HEK293FT cell medium included 400 µg/ml Geneticin (Gibco). Transient transfections were carried out 24 h after seeding 550,000 HEK293FT or 75,000 COS7 cells per well of a 6-well plate. Genejuice (Novagen) transfection reagent was used according to the manufacturer's instructions. Cells were harvested 24 h later using 0.05% Trypsin-EDTA (Gibco), and re-plated into 96-well plates at a density of 100,000 cells per well (HEK293FT) or 15,000 cells per well (COS7) in phenol red free DMEM containing 5% FCS, 16–18 hours prior to assay.

### BRET Assays

HEK293FT or COS7 cells were transiently transfected with cDNA encoding wild-type or mutant V2R fused to Rluc8 (V2R/Rluc8) and β-arrestin2 fused to Venus (β-arrestin2/Venus). In one set of experiments, cells were transfected with V2R/Rluc8 constructs and a mutant ubiquitin (ubiquitin K48,63A) fused to Venus (ubiquitin/Venus). This mutant is reported to have a reduced capacity for poly-ubiquitin chain formation, thereby avoiding the potential for multiple acceptor moieties causing quenching or interference phenomena [29]. HEK293FT cells were transfected with 0.1 µg/well of V2R/Rluc8 and 0.3 µg/well of β-arrestin2/Venus or ubiquitin/Venus, whereas COS7 cells were transfected with 0.2 and 0.6 µg/well, respectively. Cells were harvested as above, plated into white 96 well plates (Nunc) and incubated in serum free, phenol red free DMEM at 37°C, 5% CO_2_ for 2 h with 30 µM EnduRen (Promega) to ensure substrate equilibrium was reached. BRET measurements were taken at 37°C using the VICTOR Light plate reader with Wallac 1420 software (PerkinElmer). Filtered light emissions were sequentially measured at 400–475 and 520–540 nm. The BRET signal was calculated by subtracting the ratio of 520–540 nm emission over 400–475 nm emission for a vehicle-treated cell sample from the same ratio for a second aliquot of the same cells treated with agonist, as described previously [30], [31]. In this calculation, the vehicle-treated cell sample represents the background, eliminating the requirement for measuring a donor-only control sample [30], [31].

### Measurement of Fluorescence

Cells were transfected as above, harvested, and replated into black 96-well plates (PerkinElmer). Fluorescence was measured on an EnVision 2102 multilabel plate reader (PerkinElmer) using a 485/14 excitation filter, 535/25 emission filter and D505 mirror.

### Measurement of cyclic adenosine monophosphate (cAMP) production using homogeneous time-resolved fluorescence (HTRF)

Intracellular cAMP levels were measured using a HTRF cAMP dynamic 2 assay kit (CisBio Bioassays, Bagnol sur Ceze, France). Cells were transfected as described above and seeded into white 96-well microplates (Nunc) 24 h prior to assay. Cell media was removed by aspiration and replaced with 40 µl stimulation buffer (0.5 mM IBMX, 5 mM HEPES, 0.1% BSA in Hank's Balanced Salt Solution; Life Technologies) containing agonists as indicated. Cells were incubated for 30 min at 37°C and then lysed by addition of 12.5 µl of the supplied conjugate-lysis buffer containing d2-labeled cAMP, followed by 12.5 µl of conjugate-lysis buffer containing terbium cryptate-labeled anti-cAMP antibody, both reconstituted according to the manufacturer's instructions. Plates were incubated for 1 h at room temperature and time-resolved fluorescence signals were measured at 620 and 665 nm, respectively, 50 µs after excitation at 337 nm using an EnVision 2102 plate reader (PerkinElmer).

### Measurement of D-myo-inositol-1-phosphate (IP1) production using homogeneous time-resolved fluorescence (HTRF)

Intracellular IP1 levels were measured using a HTRF IP-One Tb assay kit (CisBio Bioassays, Bagnol sur Ceze, France). Cells were transfected as described above and seeded into white 96-well microplates (Nunc) 24 h prior to assay. Cell media was removed by aspiration and replaced with 40 µl stimulation buffer (as supplied, including 50 mM LiCl) containing agonists as indicated. Cells were incubated for 30 min at 37°C and then lysed by addition of 12.5 µl of the supplied conjugate-lysis buffer containing d2-labeled IP1, followed by 12.5 µl of conjugate-lysis buffer containing terbium cryptate-labeled anti-IP1 antibody, both reconstituted according to the manufacturer's instructions. Plates were incubated for 1 h at room temperature and time-resolved fluorescence signals were measured at 620 and 665 nm, respectively, 50 µs after excitation at 337 nm using an EnVision 2102 plate reader (PerkinElmer).

### Quantification of cell surface receptor expression

Cell surface HA-tagged V2Rs were quantified by fluorescence microscopy using an automated system for image acquisition (IN Cell Analyzer 1000; GE Healthcare) and validated algorithms for image analysis [32], [33]. Briefly, COS7 cells were seeded in a 6-well plate (75,000 cells/well) and transiently transfected the next day with 1 µg HA-tagged V2R cDNA. Cells were harvested 24 h later and seeded into black 96-well plates (Greiner; 8000 cells/well in triplicate wells) for 16–18 h prior to staining. Cell surface receptor staining was performed on live intact cells by incubation with primary antibody (rabbit polyclonal anti-HA (Sigma-Aldrich) diluted 1∶1000 in DMEM with 1% BSA) for 1 h at 4°C. Cells were then washed with ice-cold phosphate-buffered saline (PBS), fixed (2% paraformaldehyde in PBS for 30 min), and permeabilized (0.1% Triton X-100 in PBS for 10 min). Cells were washed three times, blocked (1 h in PBS containing 0.1% Triton X-100 and 1% BSA), and incubated for 1 h with secondary antibody (Alexa Fluor 546-conjugated goat anti-rabbit IgG at 1∶400 in PBS with 0.1% Triton X-100 and 1% BSA). Nuclei were stained using Hoechst 33258 (1 µM, 20 min). Images were acquired, collecting four fields per well with a 10× objective and triple band filter set to obtain images of 100–1000 cells (per well) in a total imaged area of 2.4 mm^2^. Image analysis was performed with IN Cell Workstation 3.7 software (GE Healthcare) using a Dual Area Object Analysis algorithm and a filter to define the proportion of positively stained cells (cell surface HA-V2R staining >10% above background). A cell surface expression index was then calculated by multiplying the proportion of stained cells (% positive cells) by their mean fluorescence intensity in arbitrary fluorescence units, as described [32], [33].

### Data Presentation and Statistical Analysis

Data were analyzed using Prism 5 graphing software (GraphPad). Sigmoidal curves were fitted to the dose-response data using non-linear regression. Statistical analysis of pEC_50_ values was carried out using one-way ANOVA followed by Tukey's multiple comparison post-test.

## Results

### V2R signaling to cAMP and functional validation of the V2R/Rluc8 fusion protein

**Figure 1:**
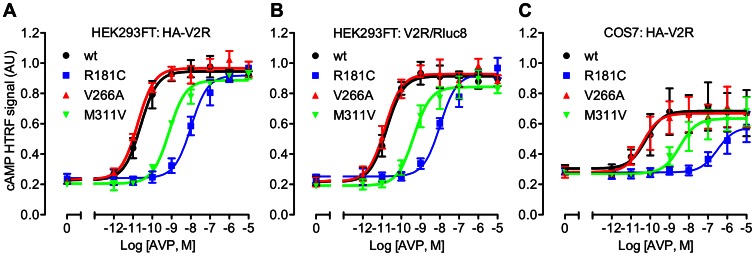
AVP-induced cAMP accumulation. Transfected HEK293FT cells (**A, B**) or COS7 cells (**C**) expressing either wild-type or mutant HA-tagged V2R (**A, C**) or Rluc8-tagged V2R (**B**), were treated for 30 minutes with stimulation buffer containing 0.5 mM IBMX together with the indicated concentrations of AVP or 50 mM forskolin. Following additions, cells were lysed and cAMP accumulation measured by HTRF. Data shown are HTRF signal in arbitrary units (AU) normalized to the forskolin response. Results shown are the mean ± SEM of 3 independent experiments.

The V2R couples to Gs protein, thereby activating adenylate cyclase and increasing intracellular cAMP. Accordingly, we tested the ability of the three mutant receptors to increase cAMP levels using a homogenous time-resolved fluorescence (HTRF) assay (Figure 1). Treatment of transiently transfected HEK293FT cells with AVP caused a robust dose-dependent increase in cAMP (Figure 1A), with particularly high potency for the wild-type and V266A mutant receptors, the pEC_50_ values of which were not significantly different (Table 1). In contrast, AVP was 29- or 427-fold less potent in cells transfected with M311V or R181C HA-V2R respectively (Table 1). Next, we tested for the ability of Rluc8-tagged V2R to couple to Gs in the same cell line (Figure 1B), as it is important to check that the addition of a BRET tag does not compromise normal receptor function [34], [35]. Again, AVP caused a dose-dependent increase in cAMP, and for each receptor, potency was comparable to that obtained with the HA-tagged V2R (Table 1). Furthermore, the ligand-induced β-arrestin recruitment shown below provides additional validation of V2R/Rluc8 functionality. We also tested whether a different cellular background influenced the effect of the mutations on cAMP signaling. No significant difference in potency was observed between wild-type and V266A mutant receptors expressed in COS7 cells compared to the HEK293FT cell line. Furthermore, a significantly lower (60-fold) AVP potency was again observed with M311V and significantly lower still with R181C (3890-fold lower than wild-type). Indeed, expression of the R181C receptor in COS7 cells resulted in a significantly lower potency of AVP compared to its expression in HEK293FT cells (Table 1).

**Table 1:** pEC_50_ values and fold change in EC_50_ for AVP-induced cAMP and IP1 accumulation.

	cAMP						IP1	
	HEK293FT				COS7		HEK293FT	
	HA-V2R		V2R/Rluc8		HA-V2R		HA-V2R	
	pEC_50_	Fold change	pEC_50_	Fold change	pEC_50_	Fold change	pEC_50_	Fold change
wt	10.57±0.27	1.0	10.78±0.14	1.0	10.10±0.17	1.0	7.82±0.13	1.0
R181C	7.94±0.20* ^,^ **	427	7.93±0.16* ^,^ **	708	6.51±0.07* ^,^ ** ^,^ ***	3891	ND	ND
V266A	10.76±0.06	0.6	10.84±0.20	0.9	10.31±0.15	0.6	7.97±0.13	0.7
M311V	9.11±0.07*	28.8	9.34±0.12*	27.5	8.32±0.25*	60.3	6.25±0.13*	37.2

**Figure 2:**
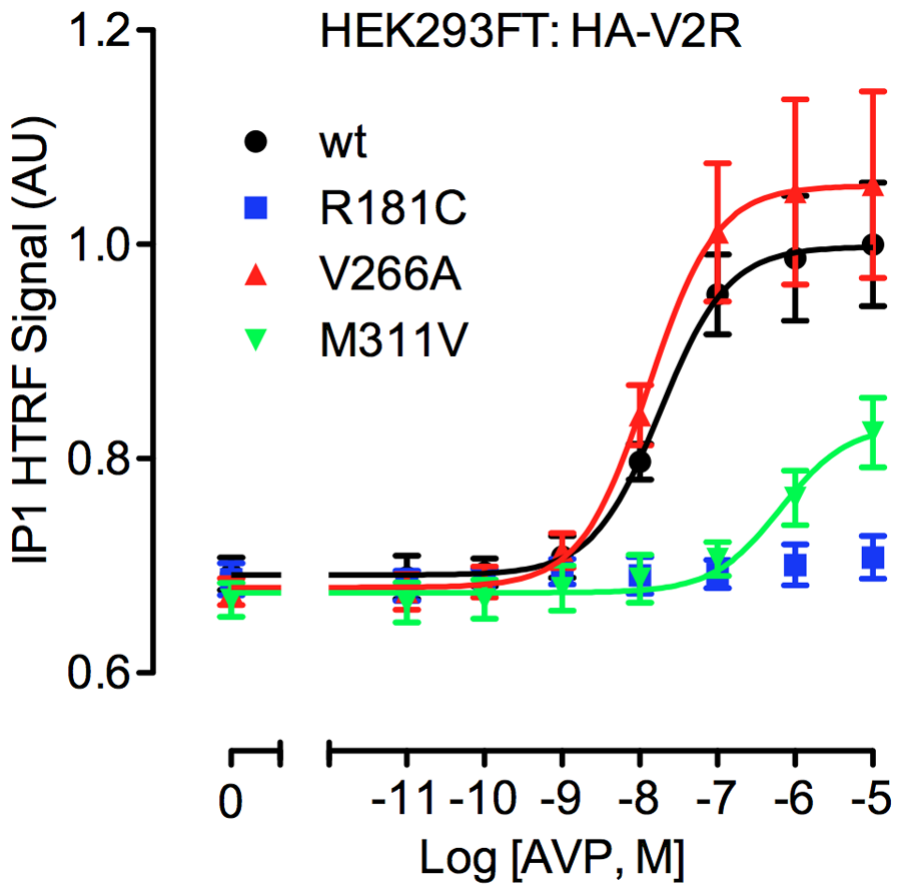
AVP-induced IP1 accumulation. Transfected HEK293FT cells expressing either wild-type or mutant HA-tagged V2R were treated for 30 minutes with the indicated concentrations of AVP. Following additions, cells were lysed and IP1 accumulation measured by HTRF. Data shown are HTRF signal in arbitrary units (AU) normalized to the wild-type receptor response. Results shown are the mean ± SEM of 5 independent experiments.

Transfected HEK293FT or COS7 cells were treated as per Figure 1 or 2. Data shown are the pEC_50_ values for AVP treatment (30 min) and are the mean ± SEM of 3 or 5 independent experiments for cAMP and IP1 respectively. Fold change from the wild-type receptor EC_50_ data are given in adjacent columns.

* , significantly different to the corresponding wild-type receptor in the same cell line (P<0.001);

** , significantly different to M311V in the same cell line (P<0.01 for HA-V2R in HEK293FT and P<0.001 for V2R/Rluc8 in HEK293FT and HA-V2R in COS7);

*** , significantly different to R181C HA-V2R in HEK293FT cells. ND = Not determined.

### Effect of V2R mutation on IP1 accumulation

Although the V2R predominantly signals through Gs, this receptor is also known to couple to the Gq class of G-proteins, activate phospholipase C and increase intracellular Ca^2+^ [36], [37]. Accordingly, we used a HTRF assay of IP1 accumulation (a downstream metabolite of inositol 1,4,5-trisphosphate; IP3) to test V2R signaling through this pathway (Figure 2). Addition of AVP to transfected HEK293FT cells caused a robust dose-dependent increase in IP1 for the wild-type receptor (Figure 2), albeit with 2–3 orders of magnitude less potency than the cAMP response (Table 1). No significant difference in IP1 accumulation was observed for the V266A receptor compared to wild-type, whereas for the M311V receptor the potency of AVP was markedly reduced (37-fold). Notably AVP failed to increase IP1 levels in cells expressing R181C V2R, even at the highest dose tested (Figure 2).

### Effect of V2R mutation on β-arrestin interaction

**Figure 3:**
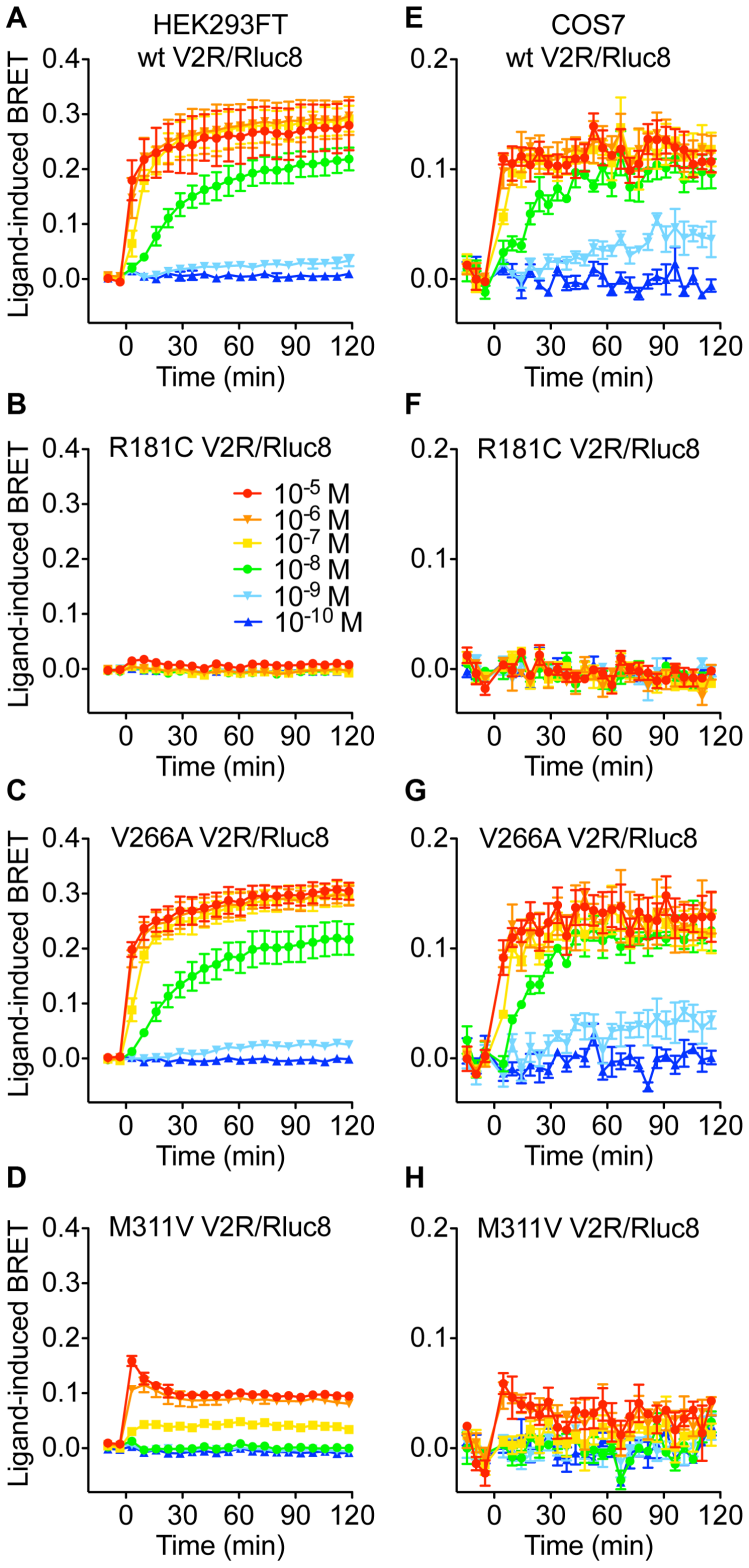
Effect of V2R mutations on the kinetics of AVP-induced β-arrestin2/Venus recruitment. Extended BRET kinetic profiles were generated with HEK293FT cells (**A, B, C, D**) or COS7 cells (**E, F, G, H**) expressing β-arrestin2/Venus with wild-type, R181C, V266A, or M311V V2R/Rluc8, as indicated. Cells were treated with indicated concentrations of AVP and kinetic BRET responses recorded. Data shown are the mean ± SEM of 3 independent experiments.

**Figure 4:**
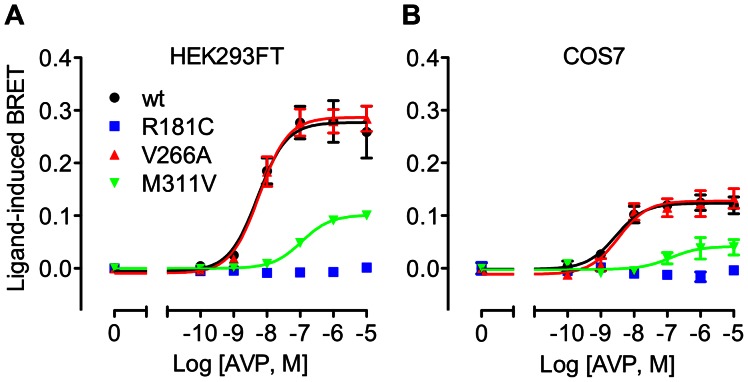
Dose-response curves for the effect of V2R mutations on AVP-induced β-arrestin2/Venus recruitment. BRET dose-response data were generated with HEK293FT cells (**A**) or COS7 cells (**B**) expressing β-arrestin2/Venus with wild-type, R181C, V266A, or M311V V2R/Rluc8. Data shown are the ligand-induced BRET response at 60 minutes post AVP treatment, and are the mean ± SEM of 3 independent experiments.

We next examined the ability of V2R/Rluc8 to interact with β-arrestin in response to AVP stimulation, using a previously validated BRET assay [13] to generate real-time kinetic profiles in both HEK293FT and COS7 cells with multiple doses of AVP (Figure 3). AVP caused a robust dose-dependent increase in BRET signal for both wild-type and V266A V2R (Figures 3 and 4, and Table 2), indicative of β-arrestin recruitment to the activated receptor. In contrast, the R181C mutant was unable to recruit β-arrestin at any dose tested, whereas the M311V receptor showed a marked reduction in both the efficacy (∼65% reduction) and potency (23-fold decrease; Table 2) of AVP. The kinetic profile with M311V V2R was also distinct from that of the wild-type receptor (Figure 3), consistent with reduced temporal stability of the receptor-arrestin complex [28]. Importantly, basal BRET ratios for wild-type and mutant V2Rs were comparable (Table 2), indicating that these mutations do not increase coupling to β-arrestin in the absence of ligand.

**Table 2:** pEC_50_ values, fold change in EC_50_, and difference in basal BRET ratio compared to wild-type for AVP-induced β-arrestin2/Venus recruitment to V2R/Rluc8.

	HEK293FT		COS7		HEK293FT	COS7
	pEC_50_	Fold change	pEC_50_	Fold change	Difference in basal BRET ratio compared to wild-type	
wt	8.31±0.21	1.0	8.54±0.02	1.0	0.000±0.008	0.000±0.009
R181C	ND	ND	ND	ND	0.014±0.008	0.000±0.003
V266A	8.24±0.19	1.2	8.45±0.05	1.2	0.020±0.012	−0.007±0.006
M311V	6.96±0.04*	22.4	7.18±0.23*	22.9	0.004±0.015	0.014±0.008

Transfected HEK293FT or COS7 cells were treated as per Figure 3. Data shown are the pEC_50_ values for AVP treatment (60 min), fold change from the wild-type EC_50_, and the BRET ratio for each receptor minus the mean BRET ratio for wild-type receptor (both prior to addition of agonist). Data are the mean ± SEM of 3 independent experiments.

* , significantly different to the corresponding wild-type receptor in the same cell line (P<0.001). ND = Not determined.

### Comparison of wild-type and mutant V2R cell surface expression

**Figure 5:**
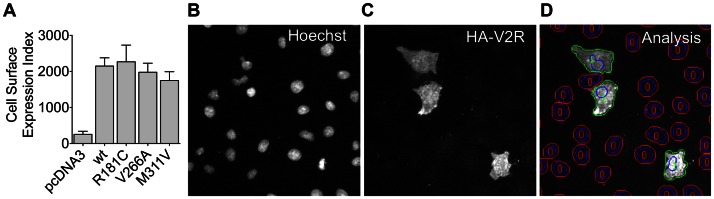
Quantification of HA-V2R cell surface expression by automated imaging. COS7 cells expressing HA-V2R constructs were stained with anti-HA antibody, initially as live intact cells, prior to image acquisition and analysis using an IN Cell Analyzer 1000. Data shown are an Expression Index defined as the percentage of positive cells (>10% above background) multiplied by their mean fluorescence intensity (**A**), and are the mean ± SEM of 3 experiments. Representative images of nuclei stained with Hoechst 33258 (**B**), cell surface HA-V2R stain (**C**), and automated image segmentation (**D**) are shown. As illustrated in (**D**), IN Cell Analyzer software was used to define perimeters of nuclei (blue) and cells (green or red) with a filter to distinguish cells in which staining was >10% above background (green perimeters (1)) or <10% above background (red perimeters (0)). Each of the image panels corresponds to a width of 250 µm and represents approximately 1/10th of the area captured in each field of view.

The majority of disease-causing mutations of the V2R exhibit a reduced ability to traffic to the plasma membrane [2]. To test for this possibility, we examined cell surface expression using a previously validated semi-automated imaging assay [32], [33]. COS7 cells expressing wild-type and mutant HA-tagged receptors were stained, initially as live intact cells, and cell surface expression determined by immunofluorescence (Figure 5). As shown, cell surface expression of each of the mutant V2Rs was comparable to wild-type. This, together with the comparable maximal ligand-induced cAMP responses (Figure 1), suggests that these receptors retain their ability to traffic to the plasma membrane. Furthermore, in both HEK293FT and COS7 cells, V2R/Rluc8 luminescence was not significantly different between wild-type and mutant V2Rs, indicating that these mutations also do not compromise overall receptor expression in each cell line (Table 3). Although no difference in expression was observed within each cell line, Rluc8-tagged receptor expression in COS7 cells was significantly lower than in HEK293FT cells (Table 3). This lower expression in COS7 cells is likely to explain the lower absolute cAMP accumulation (Figure 1) and BRET signals (Figures 3 and 4), and may contribute to the lower AVP potency with R181C V2R in COS7 compared to HEK293FT cells (Table 1).

**Table 3:** Relative V2R/Rluc8 expression in live cells.

	HEK293FT		COS7	
	RLU	% wt	RLU	% wt
wt	143394±25126	100±18	8768±3321	100±38
R181C	152886±19465	107±14	9380±3250	107±37
V266A	148174±22523	103±16	6379±1601	73±18
M311V	129322±17097	90±12	5057±1763	58±20

HEK293FT or COS7 cells were transfected with the indicated V2R/Rluc8 constructs together with β-arrestin2/Venus. Luminescence was measured in triplicate wells after 2 h pre-treatment with 30 mM EnduRen, as part of the extended BRET kinetic assays (Figure 3). Data shown are the mean ± SEM of 3–7 independent experiments. RLU = relative light units. Luminescence intensity in cells not expressing Rluc8 was 30–150 RLU.

### Determination of receptor/ubiquitin interaction

**Figure 6:**
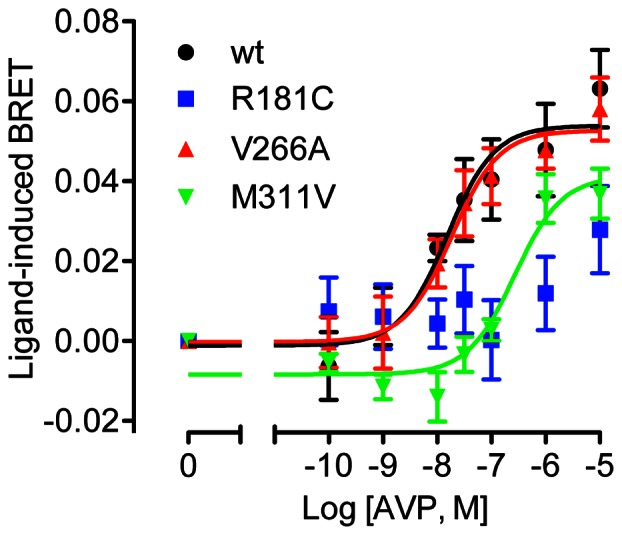
Effect of V2R mutations on AVP-induced ubiquitin/Venus recruitment. HEK293FT cells expressing ubiquitin/Venus and wild-type or mutant V2R/Rluc8 constructs were stimulated with the indicated concentrations of AVP for 120 min. Data shown are the mean ± SEM of 4 independent experiments.

The V2R undergoes agonist-induced conjugation of ubiquitin to K268, which regulates receptor degradation [38]. In a final series of experiments, we used a BRET assay of ubiquitin recruitment [29] to investigate the possibility that V266A mutation influences V2R ubiquitination, and thereby contributes to pathogenesis. As expected, AVP increased BRET between wild-type V2R/Rluc8 and ubiquitin/Venus in a dose-dependent manner (pEC_50_: 7.83±0.14) in HEK293FT cells (Figure 6). Notably, the V266A mutant displayed a very similar AVP dose-response curve (pEC_50_: 7.72±0.06) compared to the wild-type receptor. In contrast, the AVP potency with M311V V2R was significantly (P<0.001) lower (pEC_50_: 6.57±0.01) and that with R181C V2R was too low to determine, as the curve did not plateau (Figure 6).

## Discussion

In this study we have characterized the molecular pharmacology of three V2R variants from patients with differing disease phenotypes. Two of the mutants were previously described (R181C [19]–[25], and M311V [25]), and are reported to cause complete and partial NDI respectively [25]. The third variant (V266A) was found in a patient with a diagnosis consistent with NSIAD, including chronic hyponatremia and undetectable ADH levels. Using a number of functional assays we have confirmed that the M311V mutant retains greater activity than R181C V2R, consistent with the milder phenotype of NDI associated with this mutation. In contrast, the V266A V2R appears functionally identical to the wild-type receptor in all aspects of pharmacology tested by us, and others [26]. It is possible that the V266A variation requires additional modifying events, such as compound changes in G protein, β-arrestin or other proteins important for V2R signaling or regulation, for the phenotype to become penetrant. However, we feel it is more likely that the V266A change represents a rare silent polymorphism, with the causative mutation being in another protein such as aquaporin-2. This case underscores the importance of functional testing of genetic variants discovered during clinical investigations.

Of the over 200 mutations of the V2R that have been reported, at least 100 are missense mutations [8]. The majority of these mutated receptors fail to traffic to the cell surface and are retained in the endoplasmic reticulum [2]. In this regard, treatment with membrane permeable pharmacochaperones that are thought to aid in folding and functionally rescue mutant receptors may be of therapeutic benefit [18]. However, a number of V2R mutants are properly targeted to the plasma membrane, but are unable to bind AVP or signal though Gs protein to increase cAMP [2]. In this study, it appears that all three V2R variants were able to adequately traffic to the plasma membrane, as evidenced by HA-V2R staining and maximal cAMP signaling comparable to wild-type in response to AVP treatment. As such, both R181C and M311V mutants appear to be defective in ligand binding or signaling. Indeed, although to our knowledge no high-resolution structure of the V2R exists, 3D homology modeling indicates that both R181 and M311 are key residues for AVP binding [39].

The R181C mutation is present within the second extracellular loop, proximal to the plasma membrane [25]. Prior reports indicate that cysteine residue substitutions within these regions (R181C, G185C, R202C, R203C, Y205C) cause inactivating mutations and NDI [8], [25]. The V2R contains a conserved disulfide bond between C112 of the first extracellular loop and C192 of the second extracellular loop. Hence, any additional cysteine residue may interfere with formation of the conserved disulfide bond and/or introduce different deleterious disulfide bonds [40], [41].

Previous studies also report that the R181C mutant correctly traffics to the plasma membrane but has a markedly reduced capacity for AVP binding, and reduced AVP-induced cAMP production, with an approximate 1000-fold [20], 860-fold [25], or 100-fold [22] shift in potency. We observed similarly dramatic decreases in potency for cAMP accumulation in the two cell lines tested. However, the previous reports did not investigate IP1 accumulation or β-arrestin coupling for this mutant, and although high doses of AVP caused maximal cAMP responses (Figure 1), no IP1 accumulation was measured at any dose tested in HEK293FT cells (Figure 2). Similarly, no ligand-induced interaction between R181C V2R and β-arrestin was observed at any dose in either HEK293FT or COS7 cells (Figures 3 and 4). Consequently, we suggest that R181C V2R may be a Gs protein-biased receptor, albeit one for which AVP exhibits low potency compared to wild-type V2R for Gs coupling. Furthermore, with the M311V V2R, partial agonism was apparent for IP1 accumulation and β-arrestin recruitment not just a lower potency as for cAMP signaling. Although, the wild-type receptor itself exhibits approximately 2–3 orders of magnitude lower potency for AVP when signaling to IP1 and recruiting β-arrestin (pEC_50_ 7.82 and 8.31 respectively; Table 1 and 2) compared to cAMP signaling (pEC_50_ 10.57), if the R181C receptor maintained the shift observed in the cAMP assay (427-fold difference from wild-type in HEK293FT cells) one would expect to detect clear IP1 and β-arrestin responses at the highest doses of AVP tested, with theoretical pEC_50_s of approximately 5.2 and 5.7 for IP1 and β-arrestin respectively. However, no such responses are seen, suggesting a degree of Gs protein bias. Importantly as well, the potency shift for the M311V mutant is similar for IP1 and β-arrestin compared to cAMP, indicating a lack of bias in terms of potency for M311V, in contrast to R181C.

Various GPCRs have been engineered to induce signaling bias, including the dopamine D2 receptor [42], β2 adrenergic receptor [43], and designer receptors exclusively activated by designer drugs (DREADDs) [44], [45]. However naturally occurring mutations resulting in signaling bias are rare in the literature [46], particularly when the bias is towards G protein signaling. Notably, a Gs-biased thyrotropin receptor mutant has been reported [47]. This mutant is correctly expressed at the cell surface, able to increase intracellular cAMP, but markedly defective in inositol phosphate generation, resulting in an imbalance between iodide trapping (Gs-dependent) and iodide organification (Gq-dependent) [47]. Furthermore, a comprehensive examination of signaling-biased mutants was recently completed for the calcium-sensing receptor [48]. A number of biased synthetic ligands have been reported for the V2R [49], and protean agonism [50] by non-peptide antagonists was recently revealed using two V2R mutants (Ser-333del and Y128S) [51], highlighting the diverse pharmacology arising from different conformational states of the V2R.

As a result of the likely structural changes in disulfide bond formation and therefore receptor configuration discussed above, we hypothesize that the R181C mutation stabilizes a conformation even less suitable for Gq-coupling and β-arrestin interaction than Gs-coupling. This bias in ligand-induced receptor function in the absence of notable constitutive activity differs from the bias seen by Carpentier et al. with the F229V mutation, which resulted in constitutive cAMP signaling without constitutive β-arrestin recruitment, the latter function being similar to wild-type [9]. Collectively, these findings illustrate that alterations in receptor conformation due to mutation can have different effects depending on the function assessed, thus highlighting the value of comprehensively profiling multiple functions.

Following agonist stimulation the human V2R is ubiquitinated at K268, which mediates agonist-induced receptor degradation [38]. Hence we used a BRET-based assay of receptor-ubiquitin interaction to test our mutant receptors for defects in this pathway. For ubiquitin recruitment to R181C and M311V, the rank order of potency for AVP was comparable to the data obtained for intracellular cAMP accumulation (Figure 1). Interestingly, R181C retained some ability to recruit ubiquitin to the activated receptor, despite an earlier report that β-arrestin is required for this interaction [38]. Accordingly, we suggest that alternative mechanisms may be in place, or that a very weak/transient interaction of β-arrestin may suffice. Note that as both receptor-arrestin and receptor-ubiquitin assays utilized the same assay platform, it seems unlikely that our ability to detect R181C V2R ubiquitination but not β-arrestin recruitment is due to assay sensitivity.

Notably, the V266A mutation had no effect on ubiquitin interaction, in addition to wild-type-like cAMP and β-arrestin responses. The V266A variant has been previously reported in combination with a missense mutation (W200X/V266A) in a patient with NDI [26]. Here the authors also concluded that the V266A mutation has no functional consequence (as determined by radioligand binding, cAMP assay and cell surface receptor expression by ELISA), with the W200X truncation being the primary cause of NDI in that case [26]. The lack of any observable effect, shown herein and by Schulz et al, suggests that the V266A mutation may be a genetic polymorphism; one of 21 such AVPR2 gene variants reported [8]. It is important to note that the patient herein has been diagnosed with NSIAD, not NDI as in the prior study, although presumably the W200X truncation dominates over the V266A mutation when combined. Typically, NSIAD results from constitutive V2R activity (e.g. as shown for F229V [9] and R137C/L [6], [13]). However, here we find no evidence of constitutive activity. Hence, more detailed genetic studies are required to determine the precise cause of NSIAD in this patient, a starting point being aquaporin-2.

Interestingly, Zhong and colleagues engineered a V266K mutant V2R, in order to investigate potential Gq coupling properties, as a K residue at this position in the oxytocin receptor is important for IP3 generation [52]. Again, the authors report that this mutation does not influence AVP binding affinity or cAMP production. A small change (1.5 fold) in IP3 generation was reported but the authors conclude that is negligible when compared to the large (4 fold) change observed when the V2R is engineered to include the entire oxytocin receptor third intracellular loop region [52]. Notably, we found no significant difference in IP1 accumulation for the V266A mutant (Figure 2).

In conclusion, we could find no functional difference between wild-type and V266A V2R, hence the etiology of NSIAD in this patient remains unknown. We found that the M311V mutant V2R retains greater activity than R181C V2R, with respect to β-arrestin recruitment and receptor ubiquitination as well as cAMP and IP1 production, consistent with the milder phenotype of NDI associated with this mutant. Furthermore, our analysis of the R181C V2R suggests that it is a Gs protein-biased receptor. As recently discussed by Carpentier at al. [9], distinct mutations can result in distinct effects on different aspects of receptor pharmacology, and we have illustrated this in terms of both partial (M311V) and biased (R181C) agonism. Importantly, a clearer understanding of the effects of clinical mutations at the molecular pharmacological level can inform both drug discovery and patient treatment, with the tailoring of pharmaceuticals to individuals based on their receptor signaling profiles.
